# Non-Destructive Assessment of Microstructural Changes in Kabuli Chickpeas during Storage

**DOI:** 10.3390/foods13030433

**Published:** 2024-01-29

**Authors:** Navnath S. Indore, Mudassir Chaudhry, Digvir S. Jayas, Jitendra Paliwal, Chithra Karunakaran

**Affiliations:** 1 Department of Biosystems Engineering, University of Manitoba, 75 Chancellors Circle, Winnipeg, MB R3T 5V6, Canada; indoren@myumanitoba.ca (N.S.I.); mudassir.chaudhry@umanitoba.ca (M.C.); j.paliwal@umanitoba.ca (J.P.); chithra.karunakaran@lightsource.ca (C.K.); 2 President’s Office, A762 University Hall, University of Lethbridge, Lethbridge, AB T1K 3M4, Canada; 3 Canadian Light Source Inc., Saskatoon, SK S7N 2V3, Canada

**Keywords:** storage, microstructural changes, hyperspectral imaging, synchrotron X-ray imaging, chickpea

## Abstract

The potential of hyperspectral imaging (HSI) and synchrotron phase-contrast micro computed tomography (SR-µCT) was evaluated to determine changes in chickpea quality during storage. Chickpea samples were stored for 16 wk at different combinations of moisture contents (MC of 9%, 11%, 13%, and 15% wet basis) and temperatures (10 °C, 20 °C, and 30 °C). Hyperspectral imaging was utilized to investigate the overall quality deterioration, and SR-µCT was used to study the microstructural changes during storage. Principal component analysis (PCA) and Partial Least Squares Discriminant Analysis (PLS-DA) were used as multivariate data analysis approaches for HSI data. Principal component analysis successfully grouped the samples based on relative humidity (RH) and storage temperatures, and the PLS-DA classification also resulted in reliable accuracy (between 80 and 99%) for RH-based and temperature-based classification. The SR-µCT results revealed that microstructural changes in kernels (9% and 15% MC) were dominant at higher temperatures (above 20 °C) as compared to lower temperatures (10 °C) during storage due to accelerated spoilage at higher temperatures (above 20 °C). Chickpeas which had internal irregularities like cracked endosperm and air spaces before storage were spoiled at lower moisture from 8 wk of storage.

## 1. Introduction

Leguminous crops hold great significance as a nutritious food source and can assist farming systems via nitrogen fixation. The pulse production volume in Canada was around 8.8 million tonnes (Mt) in 2020, benefitting the Canadian economy by generating $1.5 billion in cash receipts. Demand for pulses has risen as they are considered protein-rich. In 2020, the pulse-seeded area in Canada was 3.5 million hectares (Mha) [1] Chickpea (*Cicer Arietinum* L.) has a rich nutritional profile with a high protein content. Kabuli type and desi chickpeas are grown in southern Canada. The country has recorded an increase in chickpea exports of 43% from 2019–2020 to 150,000 t in 2020–2021. As a result of higher demand, the average price of chickpeas of capability all types has increased by 31% to $640/t. To provide quality produce to the consumers and at trade ports, it is important to store chickpeas under optimum storage conditions. To this aim, storage studies on chickpeas were conducted [2] and a safe storage guideline chart was recommended. The determination of safe storage characteristics is conducted by monitoring the storage parameters along with abiotic and biotic factors of storage [3]. Typically, the storage temperature and moisture content (MC) are manipulated to ensure optimal conditions for seed longevity. The crops stored at higher temperatures and MC (more than 30 °C and 14%, respectively) depicted decreased germination and increased fatty acid value (FAV). Enzymatic activity, a function of high RH, affects the physical properties of the stored crops [4]. Recently, many studies have taken place to understand chickpea storage to improve its storage life and post-harvest quality [5,6,7,8].

Conventional techniques for assessing the quality of chickpeas are typically time-consuming and labour-intensive. The ‘between paper’ method is utilized to determine the germination percentage, where seeds are placed on moistened germination paper, and their sprouting is monitored over an 8-day incubation period. Moisture content (MC) is calculated on a wet basis by drying 10 g of whole chickpeas in a convection oven at 103 ± 2 °C for 72 h. The FAV (Fatty Acid Value) is measured using a Goldfisch Fat Extractor, and protein content analysis is conducted using a LECO apparatus. While effective, these traditional methods require skilled labor, are time-consuming, and often involve elaborate procedures. Considering the production and export statistics, the Canadian chickpea processing industry can significantly benefit from using rapid, reliable, environmentally friendly, cost-effective, and non-destructive quality assessment techniques [6,8,9]. These techniques can also assist in assessing chickpea quality at the ports of entry.

Hyperspectral imaging has already been well established as an effective, non-destructive technique for the quality assessment of samples in the agri-food industry. Scholars widely use two ranges in hyperspectral imaging (HSI) for crop quality evaluations, i.e., visible near-infrared (Vis-NIR) (400–1000 nm) and short-wave infrared range (SWIR) (950–2500 nm). The former spectral range is preferred for classifying the sample properties, such as colour, moisture content, soluble solid content (SSC), and acidity. In contrast, the latter range has been widely used to determine the chemical composition of the samples. These quality changes can be depicted in the crops’ spectral signatures, enabling a discriminate capability based on storage conditions. Hyperspectral imaging techniques have been used for the quality determination of flaxseed during storage, discrimination of pulse flours based on pulse type, classification of pulse flours based on milling method, determination of wheat hardness, and the characterization of cocoa beans [10,11,12,13,14]. X-rays are also becoming popular in the study of plant and food microstructures [15,16,17,18,19,20,21,22]. X-ray imaging is becoming popular due to unique characteristics such as the potential to non-destructively evaluate the growth stages and dynamic processes inside the seed, due to its capabilities, such as higher resolution, better contrast, rapid acquisition, and sensitivity to low density [23]. Synchrotron phase-contrast X-ray microcomputed tomography (SR-µCT) has become popular in the recent past in agriculture and food science applications [24,25]. SR-µCT has been extensively used to understand live dynamic processes in plants, microstructure seeds quality changes in fruits, changes in cereals during storage, and processed food products [26,27,28,29,30,31,32,33,34,35,36,37,38,39].

This research aims to simultaneously monitor the quality changes of the stored chickpeas using HSI and investigate the influence of storage parameters on the microstructure using SR-µCT. 

## 2. Materials and Methods 

### 2.1. Sample Preparation and Storage

Kabuli chickpeas (300 kg) were acquired from Reisner Farms in Limerick, Saskatchewan. The chickpeas’ moisture content (MC) at acquisition was measured to be 11% wet basis (wb). Conditioning was undertaken to attain the desired MCs of 9, 11, 13, and 15% by drying or adding a calculated amount of distilled water [6,8]. Environmental conditions of the Prairie-grown chickpeas during harvest and storage were simulated by storing the chickpeas at 10, 20, and 30 °C. Controlled relative humidity (RH) conditions were generated by using saturated salt solutions of Mg(NO_3_)_2_, NaNO_2_, NaCl, and KNO_3,_ attaining relative humidity (RHs) of 54, 65, 75, and 94% in storage, respectively. The equilibrium moisture content of chickpeas for storage at 54, 65, 75, and 94% RH was calculated as 9, 11, 13, and 15%, respectively, using the modified Henderson model and associated constants for chickpeas [40].

### 2.2. Hyperspectral Image Acquisition and Processing

**Figure 1:**
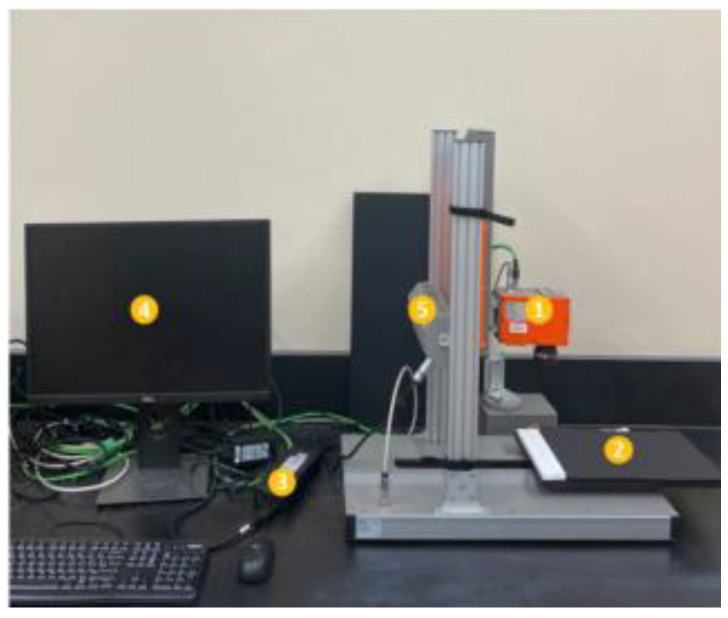
Integrated Vis-NIR I system: 1-spectrograph; 2-moving stage; 3-control panel, 4-computer system, 5-lighting system (halogen lamps).

A visible-near infrared (Vis-NIR) line-scan hyperspectral imaging system (SPECIM Spectral Imaging Ltd., Oulu, Finland) was used to acquire the images of the stored chickpea samples. The Vis-NIR camera could acquire 1024 × 896 pixel images using a spectrograph (SPECIMV 10E) and a focusing lens (SPECIM OLET 15). The Vis-NIR system operated between wavelengths of 397.66 and 1003.81 nm with a spectral resolution of 2.6 nm. For illumination purposes, two 150 W tungsten lamps (3900-ER, Illumination Technology, Inc., New York, NY, USA) were mounted at 45° over the sample platform (Figure 1). 

The vis-NIR system was turned on 30 min before the acquisition of hyperspectral images to ensure thermal and temporal stability. The frame rate for the camera was set to 20 frames per second (fps). The moving stage speed was chosen at 7 mm/s to achieve the optimum aspect ratio of frame rate and exposure duration. The hyperspectral images were corrected using the black-and-white reference images. The spectral data were extracted using the codes developed in-house in MATLAB. 

Multivariate data analysis approaches were used to investigate and interpret the trends in the extracted spectral profiles using MATLAB R2017a (version 9.2) and PLS toolbox (version 8.7.1). Principal component analysis (PCA) was used to investigate the data to determine any outliers and visualize the data trends. Principal component analysis decomposes big data with multiple correlated variables into a small number of orthogonal variables called principal components (PCs), which cover the maximum directions of variance in the data. After applying PCA, partial least squares discriminant analysis (PLS-DA) was undertaken. The PLS-DA uses non-orthogonal variables known as latent variables (LVs) to establish a co-variance between the spectral profiles and classes of dummy variables. The PLS-DA was conducted after dividing spectral datasets into calibration and independent validation sets using the Kennardstone algorithm. Spectral datasets were preprocessed utilizing mathematical pre-treatments such as mean centering, Savitzky–Golay 1st derivative, Savitzky–Golay 2nd derivative, smoothing, standard normal variate (SNV), and their combinations, before using these multivariate data analysis techniques. 

### 2.3. Synchrotron X-ray Phase-Contrast Microcomputed Tomography (SR-µCT)

Only two moisture contents samples, i.e., 9% (dry) and 15% (wet), were used in the study due to limited instrument time availability, and prior to the acquisition of the samples, they were stored in the freezer at −18 °C. The sample storage was needed until the beamtime was available, and the frozen storage does not affect the microstructure of seeds [41]. The main goal was to visualize microstructural changes inside chickpea kernels in dry and wet states and different temperatures. The imaging of the Kabuli chickpea samples was completed at the BMIT-BM beamline at the Canadian Light Source (CLS) in Saskatoon, Canada. The image acquisition was completed at 20 keV, a resolution of 3.6 µm, with a sample-to-detector distance of 5 cm. A total of 3000 projection images were captured for 180° rotation of the sample through increments of 0.06°.

Further image processing involves data normalization and reconstruction, which were completed using CLS’s in-house software, UFO-kit. The principles and theory of X-ray phase-contrast imaging have been described elsewhere [18,42,43,44]. Analysis was conducted on three individual kernels to evaluate the properties of the seed, specifically the volume and air space. A single kernel per sample was analyzed in ORS Dragonfly software (2021.3, Object Research Systems, Montreal, QC, Canada) to analyze changes due to storage deterioration. Segmentation of changes due to spoilage (and the quantification of these changes) was carried out on a single kernel that depicted the highest deterioration among three sample sets. This selection was completed by visualizing the SR-µCT data manually. 

## 3. Results and Discussion

### 3.1. PCA-Based Unsupervised Classification

**Figure 2:**
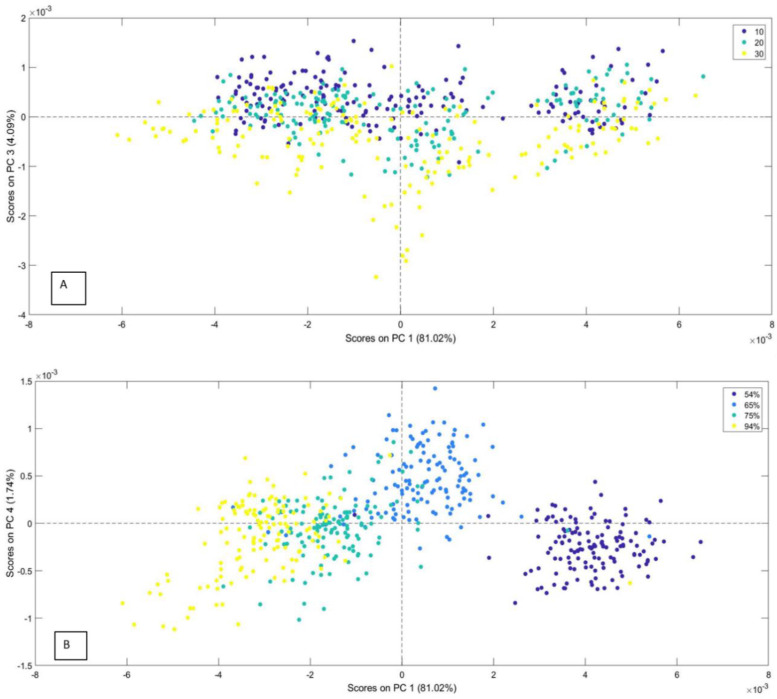
(**A**) Scores plot for the PC1 × PC3 separated by temperature (°C). (**B**) Scores plot for the PC1 × PC4 separated by Relative Humidity (RH).

The PCA modeling was completed using a spectral dataset of 537 samples in the Vis-NIR range. The most appropriate preprocessing of the Vis-NIR spectra was found to be the 1st derivative, followed by mean centering. The first principal component (PC1) covered 81.02% of the total variance; the rest, PC2, PC3, PC4, and PC5, covered 18.98%. The PCA model effectively grouped the samples based on their temperatures and RH of storage. The scores plots depicted that samples at high temperature (30 °C) and low RH (54%) were among the best discriminated. The scores plot depicted trends and clustering of different groups of RH and temperature, as shown in Figure 2. Figure 2A shows the scores plot of PC1 versus PC3 separated based on the different storage temperatures. This figure shows a separation between samples stored at 30 °C and those stored at lower temperatures along the PC3 axis. Figure 2B shows the scores plot of PC1 versus PC4, depicting a clear separation between different RH levels. PC4 assists in distinguishing the 65% RH level group from the other groups. PC1, in this case, also shows that it was hard to discriminate between the samples stored at 75% and 94% RH. However, the samples stored at 30 °C showed a rapid decrease in quality over time. In the case of the present study, it can be inferred that the clear segregation of the samples stored at 30 °C from those at lower temperatures is because of the rapid quality deterioration of chickpeas at 30 °C. 

#### PLS-DA Based Supervised Classification

The PCA results depicted that chickpeas can be grouped based on the storage temperature and RH during storage. Prior to the development of PLS-DA models for RH-based classification of chickpeas, the data were preprocessed with the 1st derivative and mean centering. Venetian blinds were used as a cross-validation method with 19 data splits. The calibration data set comprised a total of 375 samples. The PLS-DA classification model in calibration was developed using 6 LVs, explaining 86.90% of the total covariance in the data. The calibration and cross-validation performance were evaluated using sensitivity and specificity values. In this regard, the lowest sensitivity was recorded for the 75% RH class, and the highest sensitivity was observed for the 54% RH class, like the findings of the PCA model. The reliability of the calibration model was examined using an independent validation set of 162 samples. The PLS-DA prediction model showed the lowest sensitivity for the 65% RH class, as 54% of the RH class was 100% correctly classified. The classification results are elaborated in the confusion matrix in Table 1. 

In the case of classification based on the temperature of storage, 5 LVs were utilized to develop the calibration model, explaining 92.98% of the total covariance in the data. The reliability of the calibration model was examined using an external validation set of 162 samples in this case as well. The confusion matrix depicting the calibration, cross-validation, and prediction statistics is shown in Table 2. The highest sensitivity was observed for the samples stored at 10 °C, which depicts that the chickpeas went through minimal quality degradation. A large number of samples from 20 °C samples were also misclassified as 10 °C. This is because the samples did not degrade at the start of the storage period. So, the samples from the initial storage weeks 20 °C were misclassified as 10 °C. 

### 3.2. Microstructural Changes

**Figure 3:**
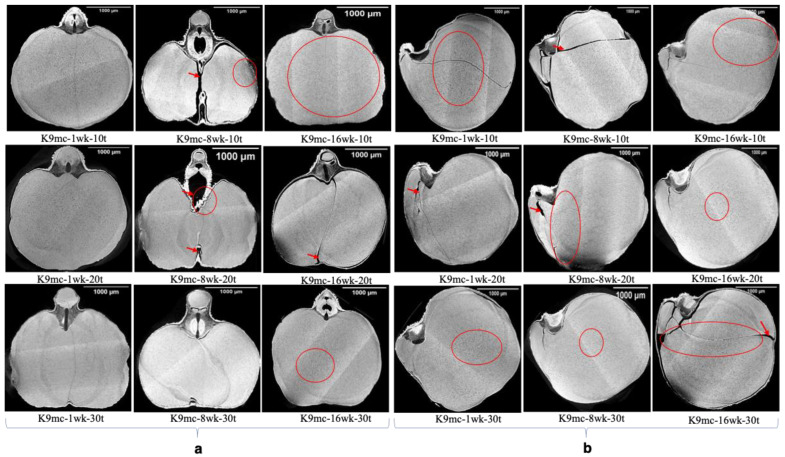
SR-µCT reconstructed data of Kabuli chickpeas stored for up to 16 wk at 9% moisture (dry) and three temperatures (10 °C, 20 °C, and 30 °C). (**a**) cross-sections (**b**) longitudinal sections (*n* = 1, single kernel was used in image processing). The red circle denotes the changes due to deterioration in the endosperm, and the red arrows indicate the air spaces/cracks. The explanation for sample names is as follows: K for Kabuli; a number preceding mc means percent mc; a number preceding wk means storage period; number preceding t means storage temperature).

**Figure 4:**
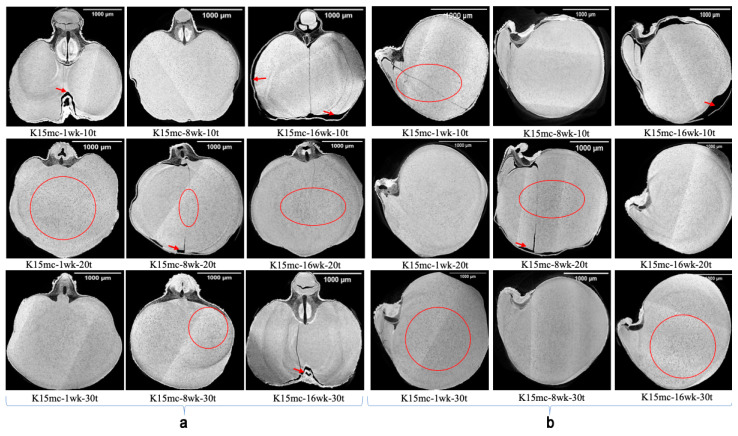
SR-µCT reconstructed data of Kabuli chickpeas stored up to 16 wk at 15% moisture (wet) and three temperatures (10 °C, 20 °C, and 30 °C). (**a**) cross-sections (**b**) longitudinal sections (*n* = 1, the single kernel was used in image processing). The red circle denotes the changes due to deterioration in the endosperm, and the red arrows denote the air spaces/cracks. The explanation for sample names is as follows: K for Kabuli; the number preceding mc means percent mc; the number preceding wk means storage period; and number preceding t means storage temperature).

**Figure 5:**
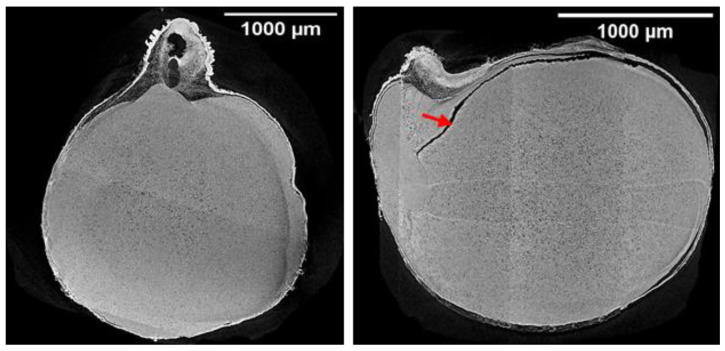
Control chickpea SR-µCT image showing existing cracks inside the kernel. The red arrow denotes the air spaces/cracks.

The results of the reconstructed projections of SR-µCT data are shown in Figure 3, Figure 4 and Figure 5. The visual observation of the projection of SR-µCT data revealed chickpeas stored at higher temperatures were more susceptible to deterioration. The storability of kernels depends on existing cracks inside the endosperm and air spaces between the coat and cotyledons, as visible in Figure 3, Figure 4 and Figure 5 of samples (control, K9mc-1wk-10t (explanation for sample names is: K for Kabuli; number preceding mc means percent mc; number preceding wk means storage period; number preceding t means storage temperature)), K9mc-8wk-10t, K9mc-1wk-20t, K9mc-16wk-30t, K15mc-1wk-10t, K15mc-16wk-10t, K15mc-8wk-20t, and K15mc-16wk-20t), which make seed microstructure vulnerable to infection due to moisture. It was found from the visual inspection of CT data that drying from initial mc to 9% mc may have induced cracks; therefore, samples stored at 15% mc had fewer cracks or air spaces. One more reason is storage time, where the 15% mc chickpea had shown the presence of air space at the end of storage (16 wk), as visible in Figure 4. The main differences in microstructural changes between Figure 3 and Figure 4 are visible from 2D projections; hence, further 3D data processing is required for the quantification of these changes in microstructure.

**Figure A1:**
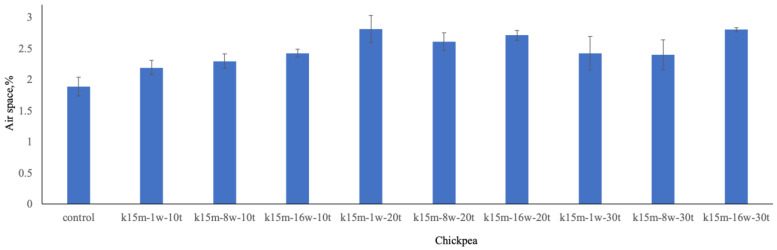
The average airspace volume in Kabuli chickpea kernels stored up to 16 weeks at 15% moisture and three temperatures (10 °C, 20 °C, and 30 °C) (*n* = 3, three kernel were used in image processing).

**Figure A2:**
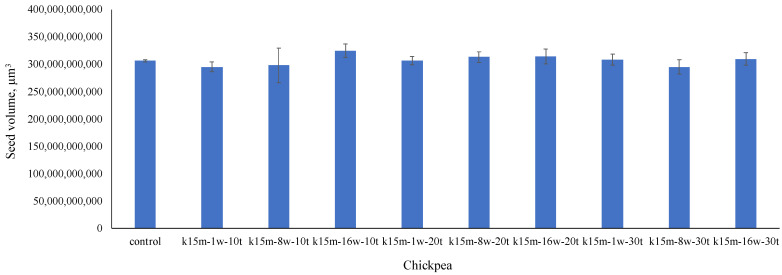
The average kernel volume of Kabuli chickpea stored for up to 16 weeks at 15% moisture and three temperatures (10 °C, 20 °C, and 30 °C) (*n* = 3, three kernel were used in image processing).

**Figure A3:**
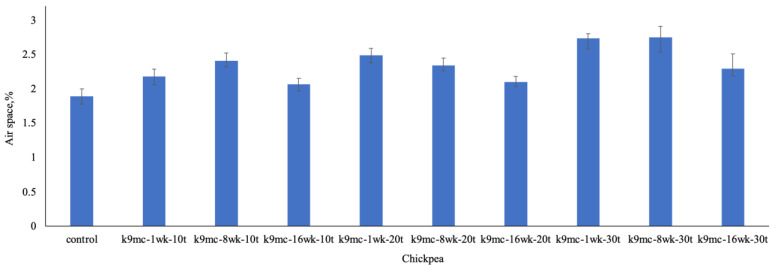
The average airspace volume in Kabuli chickpea kernels stored up to 16 weeks at moisture (9%) and three temperatures (10 °C, 20 °C, and 30 °C) [*n* = 3, three kernel were used in image processing].

**Figure A4:**
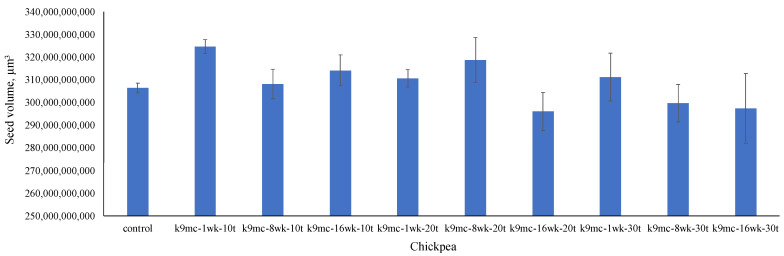
The average kernel volume of Kabuli chickpeas stored for up to 16 weeks at 9% moisture and three temperatures (10 °C, 20 °C, and 30 °C) (*n* = 3).

The change in airspace was observed for control in all treated samples during the 16-wk storage (Appendix A Figure A1 and Figure A3). The maximum changes in airspace, which includes pores and cracks, were observed in samples K9mc-8wk-10t, K9mc-1wk-30t, K9mc-8wk-30t, K15mc-8wk-10t, K15mc-1wk-20t, K15mc-16wk-20t, and K15mc-16wk-30t, with respect to control sample. Minimum changes in airspace with regard to control were observed in samples K9mc-16wk-10t, K9mc-16wk-20t, K9mc-16wk-30t, K15mc-1wk-10t, K15mc-1wk-30t, and K15mc-8wk-30t. Similarly, variations were found in seed volumes of stored samples compared to control samples, as shown in (Appendix A Figure A2 and Figure A4).

**Figure 6:**
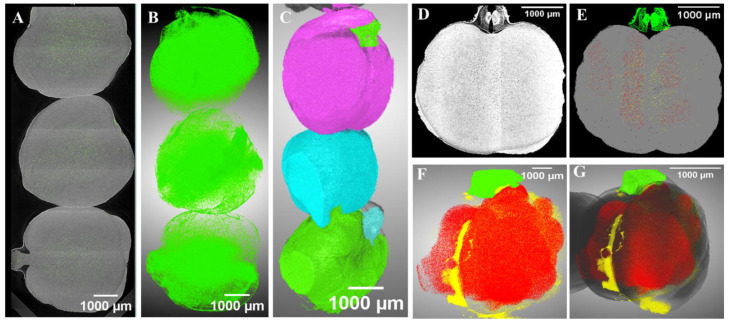
(**A**): Reconstructed vertically stitched X-ray image of three control chickpea seeds with labeled pores (green), (**B**): labeled pores in 3D visualization, (**C**): each labeled seed represents its volume, (**D**): X-ray reconstructed image of chickpea (K9mc-16wk-30t), (**E**): labeled (germ: green, change due to deterioration: red, pores: yellow and endosperm: grey), (**F**): 3D visualization of labeled microstructural features, and (**G**): full seed 3D view with all labeled features. (*n* = 1, single kernel was used in image processing).

**Figure 7:**
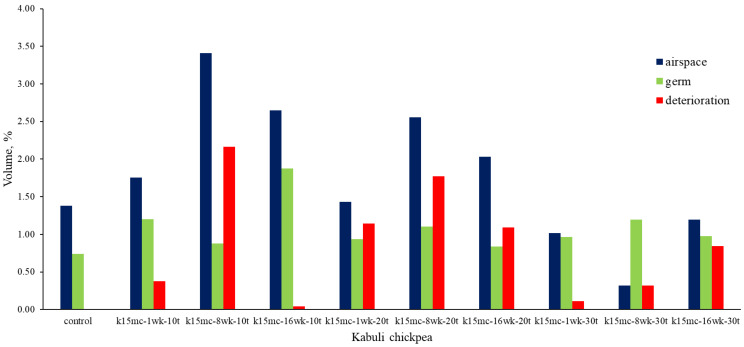
Measured volumes of segmented features of Kabuli chickpea stored for up to 16 wk at 15% moisture and three temperatures (10 °C, 20 °C, and 30 °C) [(*n* = 1, single kernel was used in image processing).

**Figure 8:**
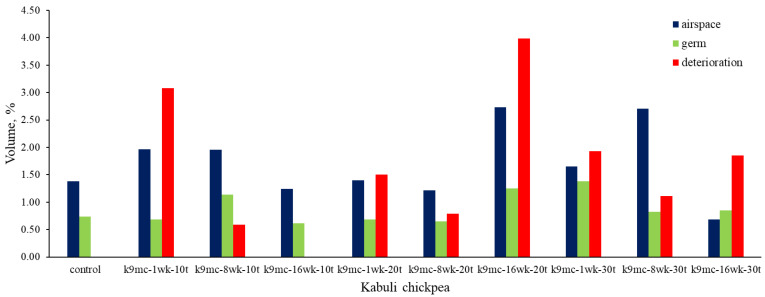
Measured volumes of segmented features of Kabuli chickpea stored up to 16 wk at 9% moisture and three temperatures (10 °C, 20 °C, and 30 °C) (*n* = 1, single kernel was used in image processing).

Detailed segmentation and measurement of microstructural changes were carried out, and the results are presented in Figure 6, Figure 7 and Figure 8. Segmentation of induced changes due to spoilage was measured from a single kernel, and here, a 3D visualization of those features is shown in Figure 6D–G in one stored sample (K9mc-16wk-30t) as an example. Figure 6A–C shows the visualization of air space and seed volume, further quantified and presented in (Appendix A Figure A1, Figure A2, Figure A3 and Figure A4).

All chickpeas stored at 9% mc showed variation in seed volume in comparison to control, but not in the case of chickpeas stored at 15% mc, where seed volumes remained unchanged for samples K15mc-1wk-20t, K15mc-8wk-20t, and K15mc-16wk-30t. This means storage conditions impact the microstructure of stored chickpeas, which might happen due to the deterioration of chickpeas, as reported earlier in the storage study [2]. More detailed measurements and visualization were carried out for quantification, as presented in Figure 7 and Figure 8. The most important part of this analysis was to highlight changes due to spoilage in chickpea kernel microstructure (endosperm, germ). It can be observed in its 3D visualization in Figure 6, where red labeled voxels represent changes due to spoilage surrounding the existing pores and cracks (marked yellow) inside the kernel and even spread inside the germ part (green voxels). 

All the 9% mc samples stored at 20 °C and 30 °C showed changes due to deterioration during storage. Still, all 15% mc chickpeas demonstrated some degree of changes due to deterioration despite a low temperature of 10 °C. In the case of 9% mc, maximum changes due to spoilage inside the kernel microstructure were found in samples K9mc-1wk-10t, K9mc-16wk-20t, K9mc-1wk-30t, and K9mc-16wk-30t. In the case of the 15% mc chickpea, maximum changes due to deterioration were found in the samples K15mc-8wk-10t, K15mc-8wk-20t, and K15mc-16wk-20t. The presence of visible mold was found in the storage study from 4 wk in the case of 15% mc, but deterioration was initiated from the first week, which was evident from SR-μCT. It was found that changes in microstructure were linked to available air space (cracks, gaps, and pores) at every storage condition, as depicted in Figure 7 and Figure 8. The size of the germ varied from 0.5 to 2% for seed volume. The length of the germ and deterioration changes inside the germ strongly correlated to a drop in germination (95% to 20%), and the lower germ volume refers to damaged or broken germ, which might happen due to material handling. Similar results were reported earlier that unfavorable storage conditions could cause microstructure changes [41]. The grain kernels’ endosperm cracking [44] was found in field samples due to weather conditions, drying, and grain handling, which affect the post-harvest quality of grains [45]. 

**Figure 9:**
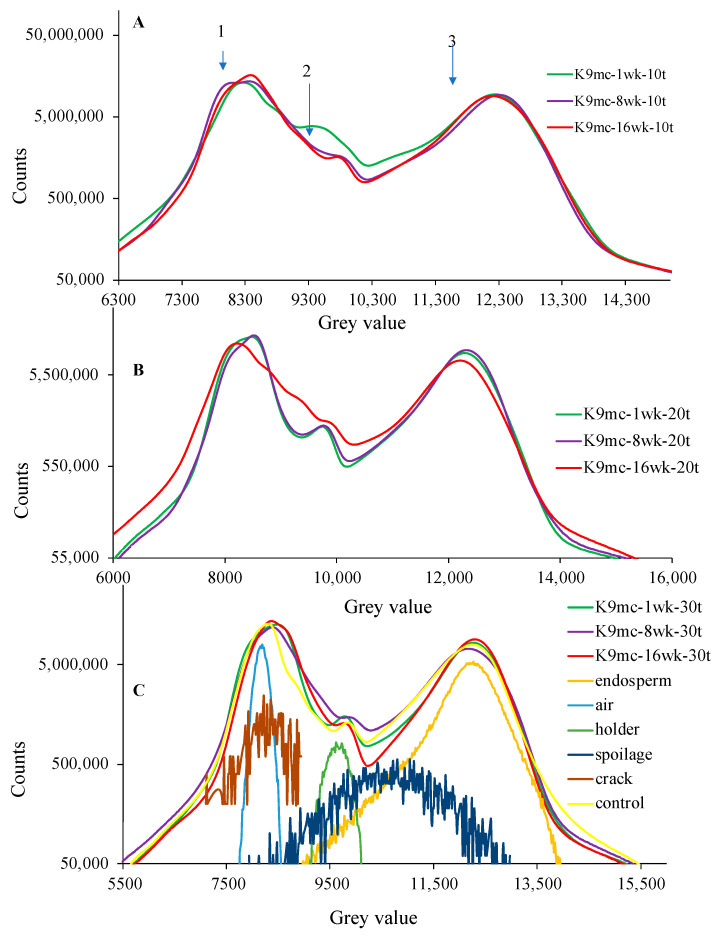
Histogram of Kabuli chickpea SR-µCT data stored at 9% mc for different weeks (1 wk, 8 wk, and 16 wk) at temperatures ((**A**): 10 °C, (**B**): 20 °C, and (**C**): 30 °C) [*n* = 3].

**Figure 10:**
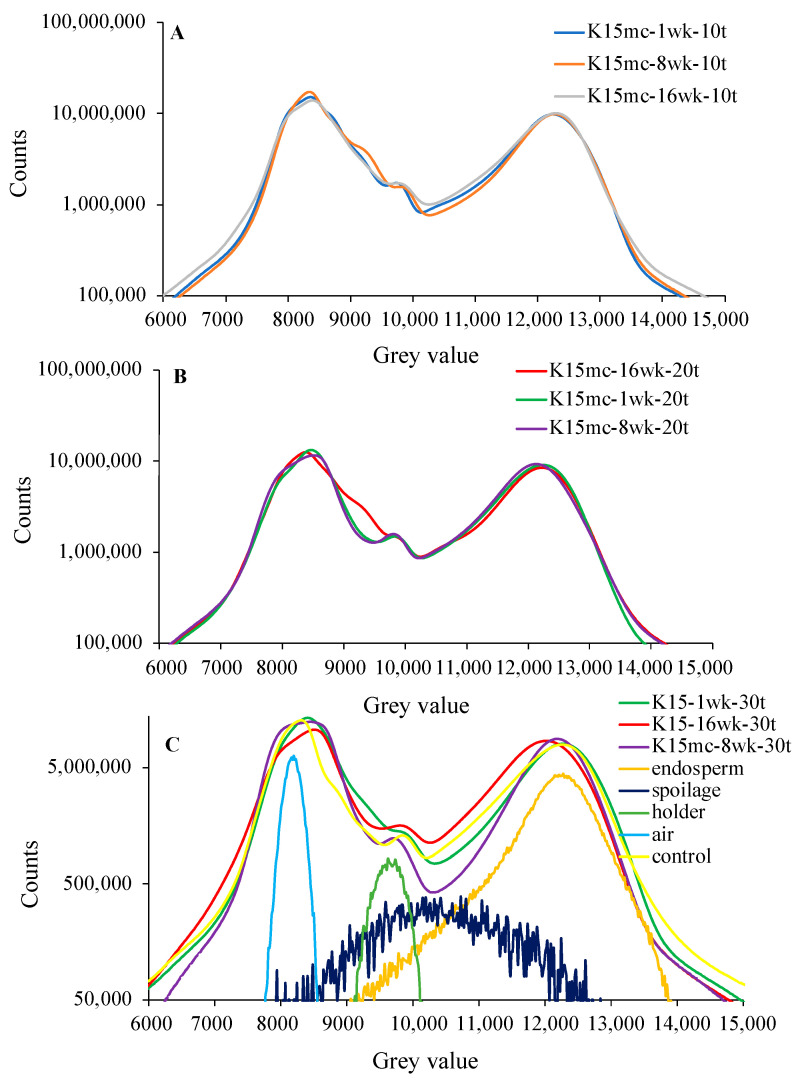
Histogram of Kabuli chickpea SR-µCT data stored at 15% mc for different weeks (1 wk, 8 wk, and 16 wk) at temperatures ((**A**): 10 °C, (**B**): 20 °C, and (**C**): 30 °C) (n = 3, three kernels were used in image processing).

The histograms of SR-µCT data were plotted for the Kabuli chickpea, as shown in Figure 9 and Figure 10. Each histogram of Figure 9A–C and Figure 10D–F represents Kabuli chickpea at 9% or 15% mc stored at different weeks at the same temperature. The first peak in the histogram represents most of the air outside the kernel, the second peak is the sample holder, and the third is the chickpea grain. The seed microstructure features such as cracks, endosperm, and change in microstructure due to spoilage in storage could be located beneath the peaks in the deconvoluted data in Figure 9C and Figure 10F. A shift in peaks was observed irrespective of lower moisture and storage time, as visible in Figure 9B,C and Figure 10E,F. The peak refers to change due to spoilage, and tilted slightly towards the left compared to the endosperm peak, and larger shifts were observed in Figure 10F. The chickpea stored at 15% mc for 16 wk at 30 °C showed a significant shift towards the left, which followed the spoilage curve underneath Figure 10F. This large shift of peaks towards the left can signify fungal damage and microstructure changes due to spoilage [18,25]. In the case of chickpeas stored at 9% mc, a noticeable change was observed at temperatures above 20 °C. The slight decrease in height in the third peak of the histogram refers to changes in volume and density, which are linked to microstructural changes. Changes in density and volume may be caused by increased storage time and temperature, as is visible in Figure 9B in 9% mc chickpeas stored for 16 wk. The presence of air space inside the kernel and cracks are responsible for spoilage during storage. The slight variation in porosity or intact seed microstructure might play a role in better performance at higher moisture, which could be inferred from Figure 9B and Figure 10E, where chickpeas stored at 9% mc at the same conditions show more variation in microstructure over 15% mc chickpea. A histogram of the control chickpea has been included in Figure 10F, which can be taken as a reference for a shift in the third peak of the chickpea stored at higher moisture and temperature at the end of storage.

The SR-μCT data revealed the microstructure information of stored chickpeas (9 and 15% mc). The 2D projections of chickpeas showed changes in gray values and variation in contrast despite having similar density across chickpea components. The SR-μCT made it possible to visualize these changes in microstructure as it is sensitive to lower density differences. Change induced by deterioration inside the seed and other constituents (air space, endosperm, seed coat) is challenging to identify due to fewer density differences when using absorption or conventional X-ray imaging. The contrast in phase-contrast imaging is achieved by varying the propagation distance between the source and the sample to the detector, eliminating the need for a contrast agent. These unique characteristics, coupled with a brighter synchrotron source, generate a high contrast in images of chickpea seeds with clear edges of components inside the seed [23]. This edge enhancement visualized the changes due to spoilage inside the endosperm having the same content. In previous chickpea storage studies, the grain quality changes were investigated by destructive methods [6,40,44,45,46,47,48,49]. This was the first attempt where a synchrotron was used to non-destructively study microstructural changes in the chickpea seed under the interaction of different storage parameters (time, moisture, and temperature). Grains can spoil faster under high moisture and temperature conditions, but actual microstructural changes can provide a better picture of what can happen at lower moisture and higher moisture under a temperature range of 10 to 30 °C. Both 2D and 3D image analyses of synchrotron data have proven the theory and result of previous chickpea storage studies [2] on the basis of microstructural changes. The histogram analysis supported the results of microstructure visualization. Chickpea, which has internal irregularities like cracked endosperm and air spaces before storage, spoiled at lower moisture during 16 wk of storage. As indicated by histogram analysis, the changes in seed volume and density of stored chickpeas were signs of deterioration, and the same was visualized in 3D image processing results. Similar results were reported in the microstructure of seeds for other food crops such as wheat, coffee, and maize [28,41,49]. The future scope of this research is to train machine learning algorithm models to identify spoilage in stored chickpeas and reduce the manual image processing time.

## 4. Conclusions

Hyperspectral imaging, in combination with multivariate data analysis techniques, successfully depicted the differences in the samples stored at different temperatures. The PCA scores plots showed the quality deterioration over time for the samples stored under different combinations of moisture, relative humidity, and temperature regimes. The PCA scores established that the fastest quality deterioration was observed for the samples stored at high temperatures (30 °C) and high initial moisture content (15%). The PLS-DA results also successfully classified the chickpeas based on their storage conditions (temperature and relative humidity). These findings suggest that this non-destructive technique can be used for tracing the samples based on their storage conditions. A notable decline in germination occurred at a higher MC of 15%. For maintaining seed quality and viability during long-term storage, storing chickpeas at MCs under 13% and temperatures below 20 °C is recommended. For the industry to effectively use non-destructive techniques, future studies should focus on the transition from hyperspectral to multispectral approaches, which are more affordable options. The synchrotron data analysis revealed microstructural changes under the interaction of different storage conditions and supported hyperspectral imaging results. Changes caused by spoilage inside stored chickpeas were visualized by edge enhancement and higher contrast of phase–contrast microcomputed tomography. The stored samples showed maximum changes in microstructure as compared to control samples. The chickpea with compromised structural integrity had changed during storage, irrespective of lower moisture of 9%. The vital components, germ, and endosperm, of chickpeas stored at higher moisture and temperature were affected, which is a sign of loss in viability. The high-resolution SR-μCT data of Kabuli chickpea seed generated in this experiment. are the first of its kind where microstructure changes inside the seed were visualized with storage time, which could help grain managers to plan their storage effectively.

## Figures and Tables

**Table 1:** PLS-DA classification statistics for relative humidity-based classification.

Calibration Results							
Prediction/Actual RH	54%	65%	75%	94%	N	Sensitivity	Specificity
54%	96	1	0	1	97	99%	99.3%
65%	0	81	8	0	97	86.6%	94.2%
75%	1	13	63	8	85	85.9%	69.3%
94%	0	2	14	87	96	95.8%	92.5%
Cross Validation Results							
Prediction/Actual RH	54%	65%	75%	94%	N	Sensitivity	Specificity
54%	96	1	0	1	97	99%	99.3%
65%	0	76	10	1	97	80.4%	93.9%
75%	1	16	58	10	85	85.9%	66.9%
94%	0	4	17	84	96	96.9%	90.3%
Prediction Results							
Prediction/Actual RH	54%	65%	75%	94%	N	Sensitivity	Specificity
54%	38	0	1	0	38	100%	99.2%
65%	0	28	4	1	38	81.6%	93.5%
75%	0	9	41	5	50	86%	59.8%
94%	0	1	4	30	36	94.4%	92.9%

**Table 2:** PLS-DA classification statistics for temperature-based classification.

Calibration Results						
Prediction/Actual (°C)	10	20	30	N	Sensitivity	Specificity
10	105	56	7	123	93.5%	67.1%
20	18	49	10	113	84.1%	48.5%
30	0	8	122	139	87.8%	94.5%
Cross Validation Results						
Prediction/Actual (°C)	10	20	30	N	Sensitivity	Specificity
10	99	60	12	123	91.9%	65.1%
20	22	42	12	113	80.5%	47.7%
30	2	11	115	139	84.2%	92.7%
Prediction Results						
Prediction/Actual (°C)	10	20	30	N	Sensitivity	Specificity
10	51	38	7	57	96.5%	48.6%
20	6	23	7	67	86.6%	31.6%
30	0	6	24	38	63.2%	92.7%

## Data Availability

High-resolution imaging data are available on request from the corresponding author, the data are not publicly available due to [part of thesis and in large files hence available on request] Additional figures are attached to the manuscript in Appendix A.
